# Duagnosis: Locating Clinical Uncertainty in the Diagnostic Framework

**DOI:** 10.1111/jep.70621

**Published:** 2026-09-25

**Authors:** Kosei Hanaoka, Daisuke Miyamori, Keiko Ogawa‐Ochiai

**Affiliations:** ^1^ General Medicine Co‐Creation Center, Kanazawa University Hospital Kanazawa Japan; ^2^ Kampo Clinical Center Hiroshima University Hospital Hiroshima Japan; ^3^ Department of General Internal Medicine Hiroshima University Hospital Hiroshima Japan

**Keywords:** clinical decision‐making, clinical reasoning, epistemology, uncertainty, medicine, Kampo, medical pluralism, medicine, traditional

## Abstract

**Rationale, Aims and Objectives:**

Clinical reasoning research has predominantly operated within a single epistemological system. The prevailing response to clinical uncertainty in primary care has been to cultivate tolerance, embrace ambiguity, or learn to navigate it: each treats uncertainty as inherent to the encounter. We propose a complementary move. Much of what is called clinical uncertainty may be attributed less to the patient's condition than to the reach of the diagnostic framework through which the clinician reasons. We introduce the concept of *duagnosis* (dual + gnosis, ‘knowing'): the clinical cognition in which two independent diagnostic systems are simultaneously activated within a single clinician's reasoning.

**Methods:**

Conceptual analysis proceeding through concept definition, differentiation from neighbouring frameworks, development of the epistemological argument, and institutional comparison across East Asia. The analysis draws on clinical reasoning research, the philosophy of medicine, evidence on the care of persistent physical symptoms, and comparative scholarship on traditional medicine.

**Results:**

The core contribution is epistemological: what one framework cannot formulate may resolve into a recognisable, actionable pattern in another, so that uncertainty is partly a property of the framework and not only of the patient. Duagnosis can be stated in two versions: a broad sense continuous with family medicine's plural readings of the patient, and a strict sense in which the criteria of evidence themselves change. An East Asian institutional comparison shows that Japan's unified medical licence locates diagnostic duality within individual clinician cognition for every physician, a capacity medical education has not cultivated.

**Conclusion:**

Expanding the diagnostic repertoire, not merely tolerating uncertainty, can reduce the proportion of presentations for which no clinical formulation exists. Locating uncertainty partly in the reach of the framework, rather than in the patient, is a reframing that applies beyond any one health system, with implications for communication and referral.

## 1 Introduction

A woman in her 30s, the mother of a 1‐year‐old, is referred to a general medicine clinic with dizziness, unsteadiness, and headaches. Postnatal checks and blood tests performed before referral have been unremarkable, as has brain magnetic resonance imaging (MRI) arranged since. Screening for postpartum depression (Edinburgh Postnatal Depression Scale score of 6) and anxiety is negative. She cannot rest: nights broken by her infant's crying, days consumed by childcare. Such presentations (real and disabling symptoms that biomedicine cannot explain, or can explain but not treat) are the daily currency of primary care and of the referrals that flow out of it.

One of us (KH) writes the following in the first person.

I have practised as a generalist physician in an urban tertiary hospital and in rural clinics. In both settings I kept meeting the same people: patients moving from physician to physician with dizziness, fatigue, loss of appetite, a painful tongue, pain in the shoulders and lower back, sensations of cold or heat, and numbness. The investigations were repeated, and each time they were told that nothing was wrong. It was the patients who kept moving. What was not reaching them, I began to suspect, was the framework.

That suspicion took shape after I trained in Kampo medicine. In the same consulting room, facing the same patients, signs I had not been seeing came into focus: the tongue, the abdominal wall, the voice. I was 3 years out of medical school; what had changed was not my clinical acumen but the framework I could bring to bear. These were not a different interpretation of the same findings; they were different findings. Sometimes a prescription followed, and sometimes it did not: seen through the Kampo framework, some patients say they feel lighter when nothing is prescribed and something concrete in their daily lives is changed. In neither case did I set aside what family medicine had taught me. I did not put one framework down to pick up the other. The order was not mine to choose: I work in hospitals built around biomedicine, and it is the biomedical account that I bring to the patient first. But by then I have already seen with both. The order was sequential; the seeing was not.

A substantial proportion of primary care patients present with medically unexplained symptoms (MUS) [1]. The prevailing response has been to frame this as a challenge of professional maturity. Simpkin and Schwartzstein asked whether tolerance of uncertainty might be ‘the next medical revolution' [2]; low tolerance has since been linked to burnout [3]. Malterud and colleagues argued for embracing uncertainty as intrinsic to general practice, the ability to act without full knowledge being a core clinical skill [4]; Gardner and colleagues found that navigating uncertainty contributes to professional identity formation [5]. Each of these responses concerns the clinician's internal capacity to tolerate, embrace, or navigate an uncertainty assumed to lie in the encounter. We ask not only how uncertainty is borne, but where it is located. Much of what we call clinical uncertainty may be less intrinsic to the patient than contingent on the diagnostic framework available to the clinician. Where uncertainty is read as a property of the patient, the patient is left carrying an unexplained complaint and the clinician has no formulation to offer; reading part of it as a property of the framework changes the question to whether another framework reaches where the first did not. Olson and colleagues argued that context is inseparable from clinical reasoning [6]. Cameron and colleagues explored how culture shapes clinical reasoning in medical education [7], and Koufidis and colleagues distinguished cognitive, situated, and socially mediated reasoning [8]. Each describes reasoning within a single diagnostic system; the question of reasoning across two independently constituted systems remains open. Separately, JECP's philosophy thematic editions have explored how epistemological assumptions define the borders of medical knowledge [9], and Lynch and colleagues named ‘Transdisciplinary Generalism' as the epistemology of generalists integrating biomedical and biographical knowledge [10]. This article continues both lines into a dimension they leave open: the diagnostic system itself as a variable in clinical reasoning. We call the cognition that works in this dimension *duagnosis*.

This argument was developed through conceptual analysis, a mode well represented in this journal [9, 10, 11]. It draws on four literature domains: clinical reasoning and uncertainty research; the philosophy and epistemology of medicine; comparative health‐systems scholarship on traditional medicine; and empirical studies of Kampo diagnostic practice. Kampo serves as the worked example for a structural reason: among traditional medical systems it holds no epistemological privilege. Several countries sustain the institutional coexistence of traditional and Western medicine. Japan is distinctive in a narrower sense. Its unified licence makes dual activation a structural possibility for every licensed physician rather than the preserve of separately trained or dual‐licensed practitioners, which makes the cognitive phenomenon observable (Section Conclusion, 4, Table 3). The clinical scenario presented is a composite derived from multiple clinical encounters, and the first‐person account describes clinical experience in general terms; no individual patient is described.

## 2 Defining Duagnosis

Returning to the case above, a physician trained in Kampo medicine (Japanese traditional herbal medicine) would observe additional signs: pallor, a weak voice, cold extremities, a pale tongue with dental indentations, and a soft, low‐tension abdominal wall on palpation. In the Kampo framework, these findings constitute a recognisable pattern: qi–blood deficiency (*ki‐ketsu‐ryōkyō*), a predictable consequence of childbirth, breastfeeding, and sleep deprivation. Where one diagnostic system runs out, another offers a clear clinical picture.

The epistemological difference is precise. In biomedicine, the same tongue pallor functions as a screening sign for anaemia; if haemoglobin is normal, the sign carries less diagnostic weight. In Kampo, tongue pallor with dental indentations may support a formulation of qi–blood deficiency independent of laboratory values. The same physical finding is deprioritised in one system and foregrounded in another.

**FIGURE 1:**
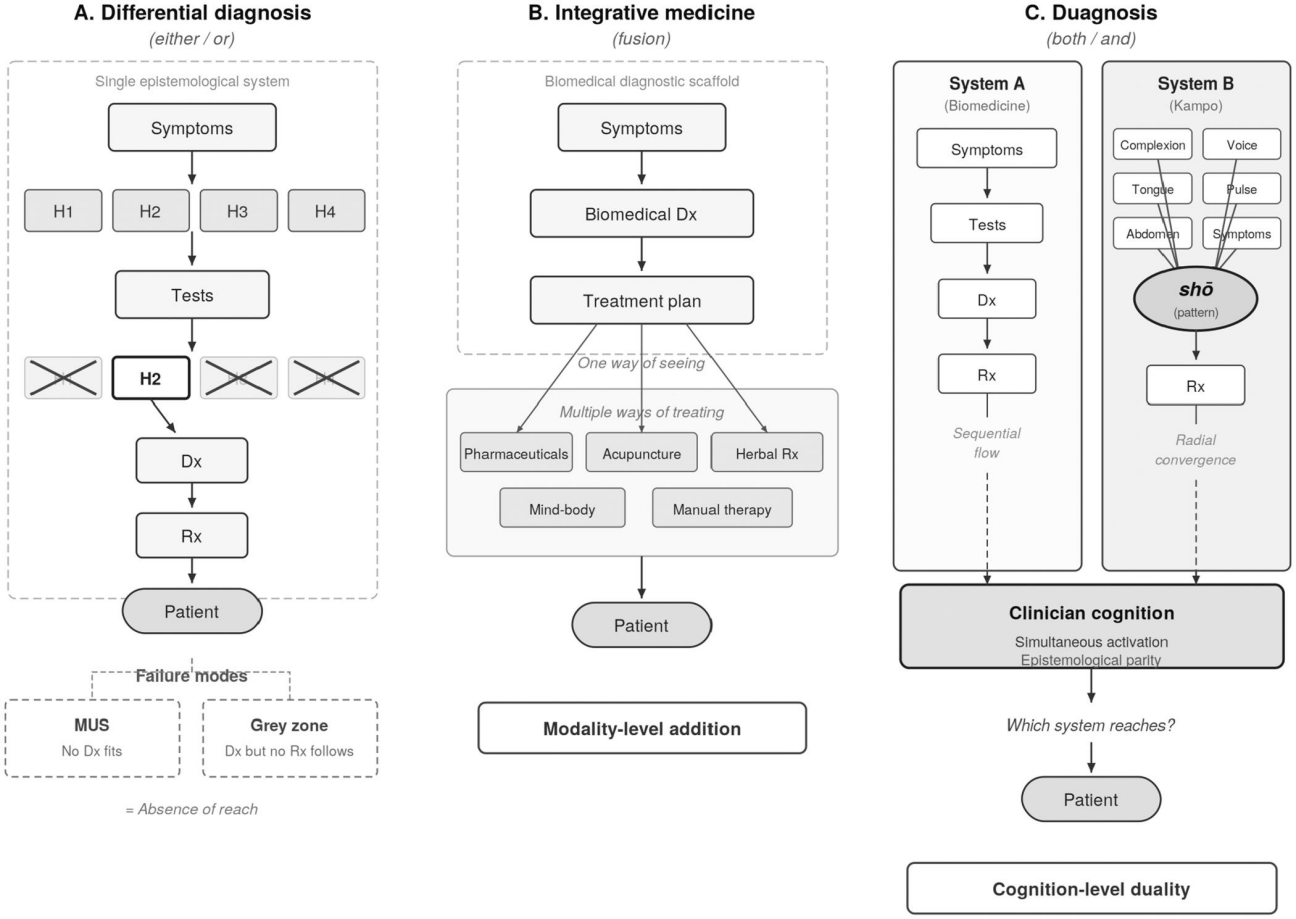
Three approaches to multiple medical systems. Differential diagnosis (either/or) narrows possibilities within a single system to converge on one diagnosis. Integrative medicine (fusion) combines therapies from multiple systems at the modality level. Duagnosis (both/and), in the strict sense distinguished in Table 2, maintains two diagnostic systems independently, activating both at the cognition level and selecting whichever pathway best reaches the patient. A single system may fail to reach the patient in two ways: no diagnosis fits (MUS) or a diagnosis fits but no treatment follows (grey zone). The two formulation processes differ structurally: System A proceeds sequentially from symptoms and tests to a diagnosis; System B converges on a holistic pattern (*shō*) from multiple findings simultaneously. In this article, System A = biomedicine (operating within a biopsychosocial framework); System B = Kampo medicine. Dx, diagnosis; MUS, medically unexplained symptoms; Rx, treatment.

We propose the term *duagnosis* (dual + *gnosis*, ‘knowing') for this clinical cognition: the concurrent operation of two independent diagnostic systems, each maintained on its own epistemological terms. The novelty of this concept lies not in the coexistence of multiple medical systems, but in the cognitive process by which two independent diagnostic systems are simultaneously activated within a single clinician's reasoning. The two formulation processes differ structurally. Biomedicine proceeds sequentially from symptoms and investigations to a diagnosis, whereas Kampo converges on a holistic pattern (*shō*) from multiple findings assessed simultaneously. The clinician determines which system ‘reaches' a given patient, or whether both contribute to the clinical formulation (Figure 1). This is not a fallback sequence. Where biomedicine formulates and treats, that formulation ordinarily leads, while the parallel seeing continues regardless of which system is acted upon. We use ‘reach' to denote a diagnostic framework's capacity to generate a formulation, meaning a clinical judgement that leads to intervention, encompassing both pattern recognition (diagnostic reach) and therapeutic action (therapeutic reach). First, duagnosis differs from *differential diagnosis*, which operates in a single epistemological framework. A psychiatrist and a neurologist may read the same symptoms differently, but both draw on biomedical ontology. In duagnosis, by contrast, each system fixes sign, pattern, and therapeutic target on its own terms.

Second, duagnosis operates at a different level from *integrative medicine*, which adds acupuncture, herbal remedies, or mind–body therapies to a biomedical treatment plan; the diagnostic scaffold remains biomedical. Duagnosis operates on the diagnostic frame itself. The question shifts from ‘what else can I offer?' to ‘can I see this patient differently through another diagnostic framework?' Prescribing a Kampo formula because a guideline recommends it for a biomedical diagnosis is still biomedical reasoning, moving from a disease name to a treatment on biomedical evidence, with the repertoire widened; recognising qi–blood deficiency from the patient's face, tongue, and pulse is reasoning of another kind, and it yields an independent clinical formulation. Duagnosis describes a cognitive act in which neither system holds default authority: epistemological parity, not hierarchical addition.

These distinctions (Table 1) let the concept be stated in two versions, differing in strictness. In a broad sense, duagnosis names a habit family medicine already has: holding more than one reading of the same patient at once, a biological and a psychosocial account, or the illness alongside the disease (Sections Results, 3 and 5). The readings differ, but they share an epistemology. The same findings count as evidence in each. In the strict sense proposed here, the readings no longer share even that. What counts as a sign changes. The tension of the abdominal wall or the quality of the pulse carries diagnostic weight in one system and none in the other. And the architecture of diagnosis changes with it. Biomedicine names an entity behind the symptoms and then infers a treatment; Kampo names a configuration of the patient in the present, from which treatment follows within the same act of recognition. Dual‐process accounts distinguish fast from slow cognition within a single epistemology [12]. Norman and colleagues argued in this journal that knowledge, not processing speed, is the central variable in diagnostic expertise [13]. If the knowledge base is epistemologically bound, a second system is not more of the same knowledge but a different kind. A precedent for this broader sense already exists inside biomedicine, and we return to it in Section Results, 3.

**TABLE 1:** Conceptual comparison: duagnosis and related frameworks.

	Differential diagnosis	Integrative medicine	Duagnosis
Level of operation	Within one epistemological system	Modality (treatment)	Epistemological framework
What varies	Diagnostic hypothesis	Treatment option	Entire diagnostic system
Number of systems	One	One + supplementary therapies	Two, independently maintained
Goal	Converge on a single diagnosis	Expand treatment options	Expand diagnostic reach

*Note:* The characterisation of integrative medicine reflects its predominant institutional practice; the field aspires to achieve multi‐system diagnostic engagement.

Both senses matter. The broad sense keeps duagnosis continuous with what generalists already do, and remains practicable where no second system is available; the strict sense is where the relocation of uncertainty becomes fully visible, because the criteria of evidence themselves change (Table 2). The two systems do not draw on the same kind of warrant: one rests on population‐level statistical inference, the other on a body of practical knowledge built and refined through generations of clinical observation.

**TABLE 2:** Two versions of duagnosis: Broad and strict senses.

	Broad sense	Strict sense
Readings held at once	More than one	More than one
Epistemology	One, shared	Two, independent
What counts as evidence	Same findings in each reading	Changes between systems
Exemplars	Biological + psychosocial account; disease + illness	Biomedicine + Kampo (or another autonomous tradition)
Institutional requirement	None	Access to a second system (e.g., a unified licence)
What it opens	Wider interpretation within one framework	A second space of findings and formulations

We describe the two systems as irreducible to each other in a pragmatic sense, not an ontological one. Whatever basic science may eventually say about the translatability of their categories, the two systems currently function as independent diagnostic frameworks with distinct rules of evidence, pattern recognition, and therapeutic decision‐making. This independence is not a placeholder awaiting future reduction; it is itself the source of clinical value.

Medical anthropology has long recognised that diagnostic categories are culturally contingent; Kleinman [14] distinguished the explanatory models held by patients and by practitioners, and located their differences in cultural and social context. Duagnosis moves from that observation to practice, treating this distinction as a trainable clinical competence.

## 3 Uncertainty as a System‐Dependent Phenomenon

We turn to the paper's central epistemological contribution: that clinical uncertainty is partly a property of the framework the clinician is reasoning inside, not solely of the patient. Han and colleagues proposed a taxonomy of uncertainty sources: probability, ambiguity, and complexity [15]. Duagnosis points toward a further dimension, the diagnostic system itself: what registers as ambiguity within one framework may resolve into a recognisable, actionable pattern within another.

The biomedical disease model can account for the mechanism (physiological depletion from childbirth, lactation, and sleep deprivation). Under Engel's biopsychosocial framework [16], general supportive measures such as rest, nutrition, and reassurance are theoretically appropriate. Yet without a somatic formulation leading to specific pharmaceutical treatment, this supportive approach often becomes watchful waiting. The problem is the absence of reach, not of explanation. This grey zone, where a biomedical explanation exists but no treatment follows, is distinct from presentations for which no biomedical explanation is available at all. In both situations, the clinician is left without a formulation that leads to action.

The two systems do not always sit easily together. A patient with persistent cold intolerance might receive a Kampo formulation for yang deficiency while still requiring thyroid investigation, and the two assessments may differ in urgency. Duagnosis does not remove the need for judgement about which assessment to prioritise; it adds a meta‐cognitive demand to coordinate across frameworks, not merely within them. When their demands conflict, excluding urgent disease keeps procedural priority; the parity duagnosis claims is epistemological, not procedural.

MUS and menopausal syndrome rank among the conditions most frequently addressed with Kampo in Japanese clinical practice [17], potentially countering the therapeutic nihilism such presentations invite.

### 3.1 The Reliability of the Second Framework

The claim is modest. Kampo diagnosis has its own ambiguities. Pattern recognition (*shō*) requires training, and inter‐clinician agreement is imperfect. It lacks the standardisation biomedicine expects of validated tools. A peer‐reviewed study of modularised *shō* pattern diagnosis reported substantial agreement for deficiency–excess classification (Gwet's AC2 = 0.71) but only fair agreement for qi–blood–fluid patterns (AC1 = 0.25) among three specialists [18], the finer‐grained distinctions likely requiring more advanced training. These ratings were made from questionnaire data with abdominal and pulse findings partially masked, so they describe the reliability of pattern assignment under restricted information rather than of full clinical examination. Treatment responses are not always predictable. The argument is not that adding a diagnostic system eliminates uncertainty, but that it reduces the proportion of presentations for which no clinical formulation exists.

Nothing in this argument displaces the priority of building evidence in the terms biomedicine recognises; duagnosis concerns what a clinician can formulate while that evidence is still incomplete. Since the clinical value of a second framework rests partly on what follows from it, Box 1 summarises the evidence base for Kampo, including its limits.

Box 1 **Evidence base for Kampo medicine.**

*A different evidentiary path*. Kampo's diagnostic core is individualised: the same biomedical condition may map to different formulations by pattern (*shō*), which sits in tension with trial designs that homogenise populations and is part of why rigorous clinical research developed later than in pharmacotherapy. The practical obstacles, including placebo manufacture for sensorially distinctive granules, have since been partly overcome, and double‐blind, placebo‐controlled trials are feasible for a subset of formulations [19]. The Japan Society for Oriental Medicine collates structured abstracts of such trials: the English‐language Evidence Reports of Kampo Treatment comprise 512 abstracts (502 randomised trials and 10 meta‐analyses) [20], and the Japanese edition has since grown to 594 [21]. Kampo formulations are recorded in 167 Japanese clinical practice guidelines (KCPG) [22], of which 44 include evidence levels and recommendation grades.

*What those trials show*. The results are modest and mixed. In functional dyspepsia, rikkunshito improved epigastric pain but did not reach its primary endpoint (*n* = 247) [23]. In the behavioural and psychological symptoms of dementia, yokukansan improved some symptoms without reaching its primary endpoint [24], with a small overall effect in an independent meta‐analysis [25]. After colonic surgery, daikenchuto showed a modest effect the authors judged insufficient to establish clinical benefit [26]. That these formulations can now be tested against placebo at all is itself the point.

*Limitations, stated plainly*. A phase III trial of goshajinkigan for oxaliplatin‐induced neuropathy was terminated after an interim analysis. Among the 182 evaluable patients, grade 2 or greater neurotoxicity was more frequent in the treatment arm than in the placebo arm [27]. A Cochrane review of daikenchuto for postoperative ileus was later withdrawn over a breach of conflict‐of‐interest policy rather than an error of science [28]. Blinding is asymmetric. Standardised granules permit matching placebos, but individually compounded decoctions, the form most tied to pattern‐based reasoning, resist concealment. Most trials have been conducted in Japan. The claim here is therefore bounded. A second framework can extend diagnostic reach, and it carries therapeutics whose benefits are real but modest and still unevenly evidenced.

### 3.2 The Scope of Therapeutic Reach

A formulation that leads to a modest intervention is still a formulation: the patient moves from an unexplained complaint to a named pattern with a treatment attached to it, and that shift has value independent of effect size. What duagnosis claims is that the patient becomes visible to a second system, not that the second system will always help.

Nor is the second system confined to cases biomedicine cannot reach. For lymphatic malformations, where treatment options are limited, a Kampo formulation was evaluated as a primary intervention in a nonrandomised clinical trial of 19 children; 10 achieved a reduction in lesion volume of 20% or more at 6 months [29]. Japan's clinical practice guidelines for vascular anomalies address Kampo formulas for this condition, concluding that their effectiveness cannot be reliably evaluated but that their use is worth considering given rare adverse reactions, a weak recommendation on weak evidence (2C) [30].

The route from a Kampo pattern to action is not always a prescription. Pattern diagnosis can issue in guidance that follows from the pattern rather than from general advice on healthy living: a patient assessed as having internal cold is advised on which foods and which forms of bathing to favour, and a patient with fluid retention (*suitai*) on how to time drinking and moving through the day, in the tradition of attending to *mibyō* (未病, ‘not‐yet‐illness'). In the first author's practice some patients improve on such guidance alone, so this clinical value does not stand or fall with the trials in Box 1.

### 3.3 Dual‐Framework Care Within Biomedicine: A Precedent

The move duagnosis proposes has a precedent inside biomedicine itself. Much of primary care already concerns symptoms that resist a purely biomedical account. Contemporary practice increasingly reframes these not as ‘non‐organic' or merely ‘medically unexplained', but as functional disorders or persistent physical symptoms, terms that patients themselves find less alienating and that carry a positive rather than an exclusionary logic [31, 32]. In functional neurological disorder, the shift from a diagnosis of exclusion to a positive diagnosis, made on the basis of characteristic signs, has itself become part of treatment: the explanation the clinician offers is therapeutic, not merely administrative [33].

Caring for such patients through both a biomedical and a psychosocial lens is not new, and the same structural logic, that widening the interpretive frame improves what can be offered, is already accepted in adjacent domains. Structured, explanation‐centred symptom clinics have improved physical‐symptom outcomes in a pragmatic trial [34], and multidisciplinary biopsychosocial rehabilitation improves pain and disability in chronic low back pain compared with usual care [35]. In this sense, holding a biological and a psychosocial reading of the same patient is duagnosis in the broad sense (Section Methods, 2).

The biopsychosocial model [16] brings its lenses to bear, on our reading, within a frame that remains biomedically anchored, its psychosocial axis adding context to a biomedical formulation rather than generating a formulation of its own; the strict sense activates two frameworks independently constituted, each with its own signs, patterns, and therapeutics. The precedent shows the direction; duagnosis extends it one epistemological step further.

The principle of *shinshin‐ichinyo* (心身一如, mind–body as one), invoked in Kampo clinical reasoning, does not separate bodily symptoms from emotional states; both are expressions of the same underlying pattern of qi–blood–fluid imbalance.

## 4 Where Duagnosis Becomes Possible: An East Asian Institutional Comparison

If uncertainty is partly system‐dependent, the next question is whether a clinician can hold two systems at once. East Asian countries share a heritage of traditional medicine rooted in classical Chinese medical theory, yet their diagnostic traditions have diverged, and their health systems have structured the relationship between traditional and Western medicine differently, with direct consequences for whether dual diagnostic cognition can occur within a single clinician.

South Korea, China, and Taiwan each institutionally separate traditional and Western medicine: dual‐track licensing of Korean Medicine in Korea [36], dedicated Traditional Chinese Medicine (TCM) universities in China [37], and more flexible dual‐licensure in Taiwan [38]. In each, dual cognition within a single clinician is prevented or confined to a dual‐licensed few (Table 3). China additionally recognises a practice scope in integrated Chinese and Western medicine, but it is entered through separate training and assessment rather than being open to every licensed physician [37, 39].

**TABLE 3:** Institutional comparison of traditional medicine in East Asia.

	Japan	China	South Korea	Taiwan
Separate TM education system for physicians	No	Yes (42 higher‐education institutions in TCM, including 25 TCM colleges, as of 2015)	Yes (12 KM universities)	Yes
Medical licensing	Unified	Dual‐track	Dual‐track (strict)	Dual‐track (flexible)
TM in national health insurance	Yes (148 Kampo formulations)	Yes	Yes	Yes
WM doctors can prescribe/use TM	Yes (all can prescribe)	Limited (permitted after training and assessment)	No (without dual licence)	Limited
TM quality preservation	Limited (48.3% bypass *shō*)	Institutionally protected	Institutionally protected	Institutionally protected
Dual cognition in one clinician	Structurally possible; educationally unachieved	Structurally difficult	Possible for dual‐licence holders (0.2% of physicians)	Possible for dual‐licence holders

Abbreviations: TM, traditional medicine; WM, Western medicine; TCM, Traditional Chinese Medicine; KM, Korean Medicine.

*Source:* Uneda et al. (2024) for the Japanese survey figures [17]; Park et al. (2012) for the cross‐country comparison [36]; the State Council Information Office (2016) for China [37]; Kim et al. (2021) for South Korea and Taiwan [38]; the Medical Practitioners Law of the People's Republic of China (2021) for the Chinese practice‐scope requirement [39]; Arai et al. (2012) [40] and Katayama et al. (2013) [41] for Japan; and Ryu et al. (2013) for the South Korean dual‐licence share [42]. The quality‐preservation row for China, South Korea and Taiwan, and the final row, are the authors' assessments derived from the arrangements above; no comparable survey of diagnostic practice exists for those systems.

The asymmetry runs deeper than institutional separation. In these systems a traditional medicine practitioner need not be a biomedical physician at all; in Japan every clinician who uses Kampo is one first, so the second framework is acquired on top of a complete biomedical training rather than alongside it.

Japan is structurally distinct. Following the Meiji‐era Medical Act of 1874, which established a qualification system for physicians [43], medical education was consolidated around Western medicine, and no university specialising in traditional Japanese medicine has since been established [40]. Chinese medicine had been developing in Japan for a millennium before it needed a name of its own; the word Kampo (‘Chinese method') was coined only in the nineteenth century, to distinguish it from the newly arrived Dutch medicine (*ranpō*, ‘Dutch method') [44, 45]. Japan thus entered the Meiji era holding two systems that were both, by name and by origin, imports.

What followed was not a negotiated boundary but an exclusion. Licensing regulations from 1883 required all practitioners to qualify in Western medicine; Kampo physicians organised societies, alliances, and repeated petitions to preserve a separate route, and in 1895 the Imperial Diet rejected their amendment by 105 votes to 78 [46]. Its twentieth‐century revival therefore had to occur inside the Western medical licence rather than alongside it. Japan's unified licence is not the product of harmony but of exclusion and a return by another route, and it is that history which places two diagnostic systems inside a single physician. Under the national health insurance, 148 standardised Kampo formulations are covered, and all licensed physicians may prescribe them [41]. Kampo education in medical schools, however, remains limited. A nationwide survey of all 80 medical schools in 2011 found that only 29% employed full‐time Kampo instructors [40], and a follow‐up survey of all 82 schools in 2019, using a near‐identical instrument, found 37%, an increase that did not reach statistical significance [47]. The result is paradoxical. A 2024 survey found that 86.7% of physicians currently prescribed Kampo medicines, yet 48.3% of prescribers did not use traditional pattern diagnosis (*shō*), the diagnostic reasoning that constitutes the cognitive core of Kampo medicine [17].

The comparison reveals a structural trade‐off (Table 3). Countries that protect traditional medicine quality through institutional separation (Korea, China, and Taiwan) thereby place the duality between practitioners rather than within one. Japan's lack of a separate traditional medicine educational system is a weakness in terms of quality preservation, but it isolates the phenomenon: diagnostic duality, when it occurs, must occur within individual clinician cognition.

## 5 Beyond Japan: Duagnosis in Context

Only the practice of duagnosis is Japan‐specific; the epistemology travels. Even where no second system is available, the reframing (‘my framework doesn't fully reach your experience' rather than ‘your symptoms are medically unexplained') changes the encounter: it may temper patients' self‐blame and clinicians' defensive practice, and sharpen onward referrals. Teaching uncertainty as having a structural and not only psychological dimension could complement existing curricula on uncertainty tolerance [5].

Duagnosis moves the questions explored in this journal's philosophy thematic editions [9] from conceptual argument to clinical practice, and extends Lynch's transdisciplinary generalism [10] to a second, independently constituted diagnostic system.

South Korea offers the closest empirical parallel for the cognitive claim. Since a 2009 change in the law, dual‐licensed physicians may practise both systems at one site, and 41.4% of those surveyed do; they lean towards biomedicine for examination and testing and towards Korean Medicine for treatment, though the tendencies are slight. What the survey found most notable was that intellectual incompatibility between the two systems was not reported as a serious difficulty, a hindrance its authors judged to diminish when one clinician holds both rather than two practitioners cooperating [42]. A separate survey of the same population found that the dual‐licensed doctors themselves judged cooperation between two clinicians impracticable and practice by one clinician more efficient [48].

### 5.1 The Epistemic Structure Beyond Kampo

What duagnosis requires of its second system is a structural property, not any particular tradition's content: an autonomous diagnostic framework, irreducible to biomedical categories, capable of generating formulations that biomedicine does not produce. Kampo and Chinese, Korean, and Indian medicine each hold this position, differing substantially in classificatory logic yet not interchangeable (Table 4). WHO's inclusion of traditional medicine in ICD‐11 (East Asian traditions as Module 1 from January 2022 [56], Ayurvedic and related systems as Module 2 released in February 2025 [57]) signals that these systems possess sufficient diagnostic systematisation to warrant formal classification. WHO states, however, that the chapter neither judges nor endorses the scientific validity of any traditional medicine practice, and confines it to optional dual coding in morbidity data rather than mortality reporting [58].

**TABLE 4:** Epistemic structure of selected traditional medical systems.

Epistemic dimension	Kampo (Japan)	Chinese medicine (China)	Korean medicine (Korea)	Ayurveda (South Asia)
Core classification	*Shō* (pattern): yin–yang, deficiency–excess, cold–heat, qi–blood–fluid; six stages of acute febrile disease	*Biànzhèng* (syndrome differentiation): the eight principles and visceral manifestation	*Sasang* constitution: four innate types (Taeyangin, Taeeumin, Soyangin, Soeumin)	*Tridoṣa*: constitution (*prakriti*) as the relative proportion of Vāta, Pitta and Kapha, fixed at conception
What treatment follows	The pattern rather than the disease name	The differentiated syndrome; therapy addresses the person rather than the illness	The constitutional type; a herb is not used across types	The constitution, which guides therapy and regimen

*Note:* This table illustrates structural parallels at the epistemic level. The classificatory content and therapeutic reasoning of each system differ substantially; the traditions listed are not interchangeable, and the comparative dimensions are the authors' analytical categories imposed for cross‐system comparison rather than categories internal to any individual tradition. What duagnosis requires is not the content of any particular tradition but a structural property: each system operates as a diagnostic framework irreducible to biomedical categories, capable of generating clinical formulations that biomedicine does not produce. Characterisations of Kampo draw on Yakubo et al. (2014) and Wu et al. (2021) [49, 50]; of Chinese medicine on the State Council Information Office (2016), Yakubo et al. (2014) and Yoo et al. (2012) [37, 49, 51]; of Korean medicine on Yoo et al. (2012) and Kim et al. (2011) [51, 52]; of Ayurveda on Prasher et al. (2008), Kurande et al. (2013) and Gupta et al. (2025) [53, 54, 55]. Comparison across several of these traditions has precedent [52].

### 5.2 Implications for Clinical Reasoning and Policy

That traditional diagnostic reasoning sits outside English‐language academic discourse is not merely a translation problem; it reflects what Neilson has called, in this journal, ‘contrasting epistemologies' [11]. Duagnosis names a capacity that works within that asymmetry rather than resolving it.

Family medicine anticipated this reframing conceptually. Its patient‐centred clinical method sets out to understand the patient as well as the disease, addressing both the physician's agenda and the patient's [59]; duagnosis extends this by bringing two frameworks to bear, rather than two agendas within one. The comprehensiveness that defines family medicine, and that improves outcomes through mechanisms which elude reductionist measurement [60], thereby acquires another dimension. If low uncertainty tolerance is associated with burnout [3], expanding the epistemic repertoire rather than merely enduring uncertainty may itself be protective.

In policy terms, the WHO Global Traditional Medicine Strategy makes the integration of traditional medicine into national health systems one of its strategic objectives [61], while the Declaration of Astana lists traditional medicines, as appropriate, alongside vaccines and diagnostics among the technologies to which access should broaden [62]. Yet the dominant model adds treatments without the diagnostic frameworks that make them coherent; Japan, where nearly half of prescribers bypass that reasoning, shows this is insufficient [17]. Duagnosis argues for integration at the level of cognition: a prescription issued without a diagnostic formulation does not reduce uncertainty.

## 6 Limitations

Duagnosis is only as useful as the second system a clinician holds, and we have examined only one. Kampo was chosen for the structural reason given in Section 5, not because its evidence base is the strongest among traditional systems. Section Results, 3 reports incomplete inter‐clinician agreement on pattern assignment, and Box 1 an evidence base whose overall methodological quality was judged low in a survey of 135 trials up to 2009 [63], though trials with more rigorous designs have since increased [19]. The cognitive cost of maintaining dual competence has not been measured, and for some clinicians it may outweigh the benefit of reduced uncertainty. The figures cited (86.7% prescribing, 48.3% non‐use of *shō*) derive from a single cross‐sectional survey and warrant replication.

The composite vignette and the first‐person account are illustrative, not evidential; the concept of duagnosis requires validation through educational intervention studies measuring diagnostic confidence and clinical outcomes. The epistemic argument of Section 5 applies to Ayurveda, mainland Chinese TCM and Korean Medicine alike, but whether the institutional conditions for practice‐level duagnosis can extend beyond a separately licensed minority, and whether holding two systems improves clinical outcomes, remain untested. The clinicians described here acquired the two systems in one order: biomedical training first. Whether the reverse order yields the same cognition is a separate question. Finally, dual‐competence training raises questions of feasibility within an already crowded curriculum, beyond the scope of this argument.

## 7 Conclusion

Clinical uncertainty is not solely a property of the patient's condition; it is partly a function of how far the clinician's framework extends. Duagnosis names the cognitive capacity this calls for.

Within Japan, the gap between widespread Kampo prescribing and the neglect of traditional pattern diagnosis represents a missed opportunity: the pharmacopoeia is integrated but the cognition is not. Beyond Japan, the reframing can inform clinical communication and referral practice wherever diagnostic uncertainty is encountered. For the case in the opening, biomedicine offered watchful waiting; Kampo provided a second formulation and a treatment that followed from it. The question duagnosis poses is whether a clinician's diagnostic framework reaches the patient, and where it does not, whether another framework can. Where one framework does not reach, the absence is not in the patient; duagnosis names the cognition that opens a second way and formulates again.

## Funding

The authors have nothing to report.

## Ethics Statement

This conceptual article does not involve human participants or patient data. No ethics approval was required.

## Conflicts of Interest

KH and DM declare no competing interests. KO‐O reports grants from Japan Agency for Medical Research and Development (AMED) and Tsumura & Co.

## Data Availability

Data sharing is not applicable to this article as no new data were created or analysed in this study.

## References

[1] M. Rosendal , T. C. Olde Hartman , A. Aamland , et al., “Medically Unexplained' Symptoms and Symptom Disorders in Primary Care: Prognosis‐Based Recognition and Classification,” BMC Family Practice 18 (2017): 18, 10.1186/s12875-017-0592-6. PMC5297117 28173764

[2] A. L. Simpkin and R. M. Schwartzstein , “Tolerating Uncertainty — The Next Medical Revolution?,” New England Journal of Medicine 375, no. 18 (2016): 1713–1715, 10.1056/NEJMp1606402. 27806221

[3] A. Simpkin Begin , M. Hidrue , S. Lehrhoff , M. G. del Carmen , K. Armstrong , and J. H. Wasfy , “Factors Associated With Physician Tolerance of Uncertainty: An Observational Study,” Journal of General Internal Medicine 37, no. 6 (2022): 1415–1421, 10.1007/s11606-021-06776-8. PMC8074695 33904030

[4] K. Malterud , A. D. Guassora , S. Reventlow , and A. Jutel , “Embracing Uncertainty to Advance Diagnosis in General Practice,” British Journal of General Practice 67, no. 659 (2017): 244–245, 10.3399/bjgp17X690941. PMC5442924 28546389

[5] N. P. Gardner , G. J. Gormley , and G. P. Kearney , “Learning to Navigate Uncertainty in Primary Care: A Scoping Literature Review,” BJGP Open 8, no. 2 (2024): BJGPO.2023.0191, 10.3399/BJGPO.2023.0191. PMC11300995 38097267

[6] A. Olson , J. E. Kämmer , A. Taher , et al., “The Inseparability of Context and Clinical Reasoning,” Journal of Evaluation in Clinical Practice 30, no. 4 (2024): 533–538, 10.1111/jep.13969. 38300231

[7] E. Cameron , H. Fleming , R. Mose , and S. Monteiro , “Exploring Context and Culture in Clinical Reasoning Medical Education: A Qualitative Exploratory Study,” Journal of Evaluation in Clinical Practice 31, no. 2 (2025): e14126, 10.1111/jep.14126. PMC11938410 39291790

[8] C. Koufidis , K. Manninen , J. Nieminen , M. Wohlin , and C. Silén , “Unravelling the Polyphony in Clinical Reasoning Research in Medical Education,” Journal of Evaluation in Clinical Practice 27, no. 2 (2021): 438–450, 10.1111/jep.13432. 32573080

[9] M. Loughlin , R. Bluhm , J. Fuller , et al., “Diseases, Patients and the Epistemology of Practice: Mapping the Borders of Health, Medicine and Care,” Journal of Evaluation in Clinical Practice 21, no. 3 (2015): 357–364, 10.1111/jep.12370. 25923823

[10] J. M. Lynch , C. Dowrick , P. Meredith , S. L. T. McGregor , and M. van Driel , “Transdisciplinary Generalism: Naming the Epistemology and Philosophy of the Generalist,” Journal of Evaluation in Clinical Practice 27, no. 3 (2021): 638–647, 10.1111/jep.13446. 32939937

[11] S. Neilson , “Contrasting Epistemologies: Biomedicine, Narrative Medicine and Indigenous Story Medicine,” Journal of Evaluation in Clinical Practice 30, no. 5 (2024): 741–748, 10.1111/jep.13914. 37526287

[12] P. Croskerry , “A Universal Model of Diagnostic Reasoning,” Academic Medicine 84, no. 8 (2009): 1022–1028, 10.1097/ACM.0b013e3181ace703. 19638766

[13] G. Norman , T. Pelaccia , P. Wyer , and J. Sherbino , “Dual Process Models of Clinical Reasoning: The Central Role of Knowledge in Diagnostic Expertise,” Journal of Evaluation in Clinical Practice 30, no. 5 (2024): 788–796, 10.1111/jep.13998. 38825755

[14] A. Kleinman , Patients and Healers in the Context of Culture: An Exploration of the Borderland Between Anthropology, Medicine, and Psychiatry (University of California Press, 1980), 104–105.

[15] P. K. J. Han , W. M. P. Klein , and N. K. Arora , “Varieties of Uncertainty in Health Care: A Conceptual Taxonomy,” Medical Decision Making 31, no. 6 (2011): 828–838, 10.1177/0272989X11393976. PMC3146626 22067431

[16] G. L. Engel , “The Need for a New Medical Model: A Challenge for Biomedicine,” Science 196, no. 4286 (1977): 129–136, 10.1126/science.847460. 847460

[17] K. Uneda , T. Yoshino , H. Ito , S. Imoto , and T. Nogami , “Current Situation and Future Issues With Kampo Medicine: A Survey of Japanese Physicians,” Traditional & Kampo Medicine 11, no. 2 (2024): 156–166, 10.1002/tkm2.1418.

[18] A. Maeda‐Minami , T. Yoshino , Y. Horiba , T. Nakamura , and K. Watanabe , “Inter‐Rater Reliability of Kampo Diagnosis for Chronic Diseases,” Journal of Alternative and Complementary Medicine 27, no. 7 (2021): 613–616, 10.1089/acm.2020.0298. PMC8290296 33861620

[19] I. Arai , “Clinical Studies of Traditional Japanese Herbal Medicines (Kampo): Need for Evidence by the Modern Scientific Methodology,” Integrative Medicine Research 10, no. 3 (2021): 100722, 10.1016/j.imr.2021.100722. PMC8181179 34136346

[20] Task Force for Evidence Reports, Committee for EBM, The Japan Society for Oriental Medicine . Evidence Reports of Kampo Treatment 2019: 512 Randomized Controlled Trials (EKAT 2019). Ver. 1.0. Tokyo: The Japan Society for Oriental Medicine; 2021, https://www.jsom.or.jp/medical/ebm/ere/index.html.

[21] Task Force for Evidence Reports, Committee for EBM, The Japan Society for Oriental Medicine . Evidence Reports of Kampo Treatment 2025 (EKAT 2025). Tokyo: The Japan Society for Oriental Medicine; 2026, https://www.jsom.or.jp/medical/ebm/er/index.html.

[22] Task Force for Clinical Practice Guidelines, Committee for EBM, The Japan Society for Oriental Medicine . Clinical Practice Guidelines Containing Kampo Products in Japan (KCPG 2025). Tokyo: The Japan Society for Oriental Medicine; 30 October 2025, https://www.jsom.or.jp/medical/ebm/cpg/index.html.

[23] H. Suzuki , J. Matsuzaki , Y. Fukushima , et al., “Randomized Clinical Trial: Rikkunshito in the Treatment of Functional Dyspepsia: A Multicenter, Double‐Blind, Randomized, Placebo‐Controlled Study,” Neurogastroenterology & Motility 26, no. 7 (2014): 950–961, 10.1111/nmo.12348. 24766295

[24] K. Furukawa , N. Tomita , D. Uematsu , et al., “Randomized Double‐Blind Placebo‐Controlled Multicenter Trial of Yokukansan for Neuropsychiatric Symptoms in Alzheimer's Disease,” Geriatrics & Gerontology International 17, no. 2 (2017): 211–218, 10.1111/ggi.12696. 26711658

[25] S. Matsunaga , T. Kishi , and N. Iwata , “Yokukansan in the Treatment of Behavioral and Psychological Symptoms of Dementia: An Updated Meta‐Analysis of Randomized Controlled Trials,” Journal of Alzheimer's Disease 54, no. 2 (2016): 635–643, 10.3233/JAD-160418. 27497482

[26] H. Katsuno , K. Maeda , T. Kaiho , et al., “Clinical Efficacy of Daikenchuto for Gastrointestinal Dysfunction Following Colon Surgery: A Randomized, Double‐Blind, Multicenter, Placebo‐Controlled Study (JFMC39‐0902),” Japanese Journal of Clinical Oncology 45, no. 7 (2015): 650–656, 10.1093/jjco/hyv056. PMC4485603 25972515

[27] E. Oki , Y. Emi , H. Kojima , et al., “Preventive Effect of Goshajinkigan on Peripheral Neurotoxicity of FOLFOX Therapy (GENIUS Trial): A Placebo‐Controlled, Double‐Blind, Randomized Phase III Study,” International Journal of Clinical Oncology 20, no. 4 (2015): 767–775, 10.1007/s10147-015-0784-9. 25627820

[28] N. Hoshino , T. Takada , K. Hida , et al., “Withdrawn: Daikenchuto for Reducing Postoperative Ileus in Patients Undergoing Elective Abdominal Surgery,” Cochrane Database of Systematic Reviews 3, no. 3 (2020): CD012271, 10.1002/14651858.CD012271.pub3. PMC7094795 32212387

[29] K. Ogawa‐Ochiai , S. Sakai , I. Saeki , et al., “Eppikajutsuto for Treatment of Lymphatic Malformations in Children: A Nonrandomized Clinical Trial,” JAMA Network Open 8, no. 11 (2025): e2540897, 10.1001/jamanetworkopen.2025.40897. PMC12584033 41182768

[30] Y. Kinoshita , K. Ishikawa , S. Akita , et al., “Japanese Clinical Practice Guidelines for Vascular Tumors, Vascular Malformations, Lymphatic Malformations, and Lymphangiomatosis 2022,” supplement, Journal of Plastic and Reconstructive Surgery 5, no. S1 (2026): 1, 10.53045/jprs.2025-0205. PMC13075649 41982806

[31] J. Stone , W. Wojcik , D. Durrance , et al., “What Should We Say to Patients With Symptoms Unexplained by Disease? The ‘Number Needed to Offend,” BMJ 325, no. 7378 (2002): 1449–1450, 10.1136/bmj.325.7378.1449. PMC139034 12493661

[32] E. M. Marks and M. S. Hunter , “Medically Unexplained Symptoms: An Acceptable Term?,” British Journal of Pain 9, no. 2 (2015): 109–114, 10.1177/2049463714535372. PMC4616968 26516565

[33] J. Stone , “Functional Neurological Disorders: The Neurological Assessment as Treatment,” Practical Neurology 16, no. 1 (2016): 7–17, 10.1136/practneurol-2015-001241. 26715762

[34] C. Burton , C. Mooney , L. Sutton , et al., “Effectiveness of a Symptom‐Clinic Intervention Delivered by General Practitioners With an Extended Role for People With Multiple and Persistent Physical Symptoms in England: The Multiple Symptoms Study 3 Pragmatic, Multicentre, Parallel‐Group, Individually Randomised Controlled Trial,” Lancet 403, no. 10444 (2024): 2619–2629, 10.1016/S0140-6736(24)00700-1. 38879261

[35] S. J. Kamper , A. T. Apeldoorn , A. Chiarotto , et al., “Multidisciplinary Biopsychosocial Rehabilitation for Chronic Low Back Pain,” Cochrane Database of Systematic Reviews 2014, no. 9 (2014): CD000963, 10.1002/14651858.CD000963.pub3. PMC10945502 25180773

[36] H. L. Park , H. S. Lee , B. C. Shin , et al., “Traditional Medicine in China, Korea, and Japan: A Brief Introduction and Comparison,” Evidence‐Based Complementary and Alternative Medicine 2012 (2012): 429103, 10.1155/2012/429103.

[37] State Council Information Office of the People's Republic of China ., Traditional Chinese Medicine in China [White Paper] (State Council Information Office, 2016).

[38] D. Kim , C. C. Shih , H. C. Cheng , S. H. Kwon , H. Kim , and B. Lim , “A Comparative Study of the Traditional Medicine Systems of South Korea and Taiwan: Focus on Administration, Education and License,” Integrative Medicine Research 10, no. 3 (2021): 100685, 10.1016/j.imr.2020.100685. PMC7903058 33665088

[39] Medical Practitioners Law of the People's Republic of China . Adopted at the 30th Session of the Standing Committee of the 13th National People's Congress, 20 August 2021; effective 1 March 2022. Official English translation. Beijing: National People's Congress, http://en.npc.gov.cn.cdurl.cn/2021-08/20/c_875935.htm.

[40] M. Arai , S. Katai , S. Muramatsu , T. Namiki , T. Hanawa , and S. Izumi , “Current Status of Kampo Medicine Curricula in All Japanese Medical Schools,” BMC Complementary and Alternative Medicine 12 (2012): 207, 10.1186/1472-6882-12-207. PMC3528449 23122050

[41] K. Katayama , T. Yoshino , K. Munakata , et al., “Prescription of Kampo Drugs in the Japanese Health Care Insurance Program,” Evidence‐Based Complementary and Alternative Medicine 2013 (2013): 576973, 10.1155/2013/576973. PMC3914391 24550992

[42] J. Ryu , B. Choi , B. Lim , S. Kim , and Y. Yun , “Medical Practices and Attitudes of Dual‐Licensed Medical Doctors in Korea,” Evidence‐Based Complementary and Alternative Medicine 2013 (2013): 183643, 10.1155/2013/183643.

[43] H. Onishi , “History of Japanese Medical Education,” Korean Journal of Medical Education 30, no. 4 (2018): 283–294, 10.3946/kjme.2018.103. PMC6288623 30522257

[44] K. Kuchta and S. Cameron , “Editorial: Kampo Medicine in a Modern Context: Ethnopharmacological Perspectives,” Frontiers in Pharmacology 13 (2022): 971254, 10.3389/fphar.2022.971254. PMC9480492 36120328

[45] K. Kuchta , “Traditional Japanese Kampo Medicine – History of Ideas and Practice; Part 1: From Ancient Shamanic Practice to the Medical Academies of Edo,” Traditional & Kampo Medicine 6, no. 2 (2019): 49–56, 10.1002/tkm2.1209.

[46] R. Shibata , G. X. Chu , X. Lin , and J. R. Fu , “Development and Future Trends of Traditional Kampo Medicine in Japan,” Chinese Medicine and Culture 5, no. 1 (2022): 58–64, 10.1097/MC9.0000000000000001.

[47] T. Nogami , M. Arai , T. Ishigami , et al., “Comparison of the 2011 and 2019 Kampo Medicine Curricula Across All Japanese Medical Schools,” Tokai Journal of Experimental and Clinical Medicine 46, no. 2 (2021): 75–82. 34216479

[48] J. Lim , Y. Yun , S. Lee , Y. Cho , and H. Chae , “Perspectives on Medical Services Integration Among Conventional Western, Traditional Korean, and Dual‐Licensed Medical Doctors in Korea,” Evidence‐Based Complementary and Alternative Medicine 2013 (2013): 105413, 10.1155/2013/105413. PMC3870632 24382975

[49] S. Yakubo , M. Ito , Y. Ueda , et al., “Pattern Classification in Kampo Medicine,” Evidence‐Based Complementary and Alternative Medicine 2014 (2014): 535146, 10.1155/2014/535146. PMC3950553 24701241

[50] X. Wu , T. K. Le , A. Maeda‐Minami , et al., “Relationship Between Conventional Medicine Chapters in ICD‐10 and Kampo Pattern Diagnosis: A Cross‐Sectional Study,” Frontiers in Pharmacology 12 (2021): 751403, 10.3389/fphar.2021.751403. PMC8721141 34987389

[51] J. Yoo , E. Lee , C. Kim , J. Lee , and L. Lao , “Sasang Constitutional Medicine and Traditional Chinese Medicine: A Comparative Overview,” Evidence‐Based Complementary and Alternative Medicine 2012 (2012): 980807, 10.1155/2012/980807.

[52] J. Y. Kim , D. D. Pham , and B. H. Koh , “Comparison of Sasang Constitutional Medicine, Traditional Chinese Medicine and Ayurveda,” Evidence‐Based Complementary and Alternative Medicine 2011 (2011): 239659, 10.1093/ecam/neq.052. PMC3143585 21949669

[53] B. Prasher , S. Negi , S. Aggarwal , et al., “Whole Genome Expression and Biochemical Correlates of Extreme Constitutional Types Defined in Ayurveda,” Journal of Translational Medicine 6 (2008): 48, 10.1186/1479-5876-6-48. PMC2562368 18782426

[54] V. Kurande , A. E. Bilgrau , R. Waagepetersen , E. Toft , and R. Prasad , “Interrater Reliability of Diagnostic Methods in Traditional Indian Ayurvedic Medicine,” Evidence‐Based Complementary and Alternative Medicine 2013 (2013): 658275, 10.1155/2013/658275. PMC3803118 24191170

[55] A. Gupta , V. Singh , S. Chandra , and R. Garg , “Towards Standardization of Prakriti Evaluation: A Scoping Review of Modern Assessment Tools and Their Psychometric Properties in Ayurvedic Medicine,” Journal of Ayurveda and Integrative Medicine 16, no. 4 (2025): 101157, 10.1016/j.jaim.2025.101157. PMC12269270 40582042

[56] World Health Organization . ICD‐11 for Mortality and Morbidity Statistics: Supplementary Chapter Traditional Medicine Conditions. Geneva: World Health Organization; 2022, https://icd.who.int.

[57] S. R. Thrigulla , V. K. Lavaniya , V. R. Narayanan , et al., “India's Roadmap for ICD‐11 TM2 Implementation: Integrating Ayurveda, Siddha, and Unani into Global Health Standards,” International Journal of Ayurveda Research 6, no. 4 (2025): 399–407, 10.4103/ijar.ijar_337_25.

[58] World Health Organization . Frequently Asked Questions: Traditional Medicine [Internet]. Geneva: World Health Organization; [cited 24 August 2026], https://www.who.int/standards/classifications/frequently-asked-questions/traditional-medicine.

[59] J. H. Levenstein , E. C. McCracken , I. R. McWhinney , M. A. Stewart , and J. B. Brown , “The Patient‐Centred Clinical Method. 1. A Model for the Doctor‐Patient Interaction in Family Medicine,” Family Practice 3, no. 1 (1986): 24–30, 10.1093/fampra/3.1.24. 3956899

[60] K. C. Stange and R. L. Ferrer , “The Paradox of Primary Care,” Annals of Family Medicine 7, no. 4 (2009): 293–299, 10.1370/afm.1023. PMC2713149 19597165

[61] World Health Organization . Global Traditional Medicine Strategy 2025–2034. Geneva: World Health Organization; 2025, https://www.who.int/publications/i/item/9789240113176. 10.2471/BLT.25.293414 PMC12578523 41180270

[62] World Health Organization, UNICEF ., Declaration of Astana (Geneva: World Health Organization, 2018).

[63] K. Watanabe , K. Matsuura , P. Gao , et al., “Traditional Japanese Kampo Medicine: Clinical Research Between Modernity and Traditional Medicine—The State of Research and Methodological Suggestions for the Future,” Evidence‐Based Complementary and Alternative Medicine 2011 (2011): 513842, 10.1093/ecam/neq.067. PMC3114407 21687585

